# Which Method to Use for Surgical Ablation of Atrial Fibrillation Performed Concomitantly with Mitral Valve Surgery: Radiofrequency Ablation *versus* Cryoablation

**DOI:** 10.21470/1678-9741-2018-0130

**Published:** 2018

**Authors:** Ünsal Vural, Ahmet Yavuz Balcı, Ahmet Arif Ağlar, Mehmet Kızılay

**Affiliations:** 1 Department of Cardiovascular Surgery, Dr. Siyami Ersek Thoracic and Cardiovascular Surgery Training and Research Hospital, Istanbul, Turkey.

**Keywords:** Mitral Valve/surgery, Cryosurgery, Catheter Ablation, Ablation, Ablation Techniques

## Abstract

**Objective:**

The effects of energy source on the maintenance of sinus rhythm and the contribution of demographic characteristics to the case selection in patients submitted to ablation performed concurrently with mitral valve surgery were analyzed.

**Methods:**

Cryothermal (n=42; 43.8%) and radiofrequency (n=54; 56.3%) energy were employed in 96 patients submitted to mitral valve replacement and Cox maze IV procedure. Patients were called for control visits between 15 days and 12 months after discharge. The causal relationship between recurrence of atrial fibrillation and factors such as left atrial diameter, C-reactive protein, hypertension, left ventricular ejection fraction, chronic obstructive pulmonary disease, and body mass index was determined.

**Results:**

Maintenance rates of the sinus rhythm with radiofrequency and cryoablation were 97.6% and 96.3%, respectively, in the first postoperative month, whereas at the 12^th^ postoperative month were 88.1% and 83.3%. No significant difference was found between groups in relation to the energy source. Sensitivity and specificity for left atrial diameter with a cut-off value of 50.5 mm were 85.7% and 70.7%, respectively. Sensitivity and specificity for C-reactive protein with a cut-off value of 12 mg/dL on the 15^th^ postoperative day were 83.3% and 88.9%, respectively. The effect of body mass index on atrial fibrillation recurrence was 3.2 times. Sensitivity and specificity for left ventricular ejection fraction 37% cut-off value were 96.3% and 11.4%, respectively. Atrial fibrillation in hypertensive cases was 5.3 times more. In patients with chronic obstructive pulmonary disease, recurrence of atrial fibrillation was 40%. The causal relation between recurrence of atrial fibrillation and the studied factors was established.

**Conclusion:**

Demographic characteristics have a significant impact on ablation efficiency, while the type of energy source does not.

**Table:** 

Abbreviations, acronyms & symbols				
**AF**	**= Atrial fibrillation**		**ECG**	**= Electrocardiogram**
**ACC**	**= American College of Cardiology**		**EHRA**	**= European Heart Rhythm Association**
**AHA**	**= American Heart Association**		**ESC**	**= European Society of Cardiology**
**BMI**	**= Body mass index**		**HRS**	**= Heart Rhythm Society**
**COPD**	**= Chronic obstructive pulmonary disease**		**LVEF**	**= Left ventricular ejection fraction**
**CrA**	**= Cryothermal ablation**		**RFA**	**= Radiofrequency ablation**
**CRP**	**= C-reactive protein**		**ROC**	**= Receiver operating characteristic**
**ECAS**	**= European Cardiac Arrhythmia Society**			

## INTRODUCTION

Atrial fibrillation (AF) is the most common cause of arrhythmia and its incidence increases with age. The prevalence in developed countries is 1.5-2%. It reaches 17% in advanced age and 80% for mitral valve disease^[^^1^^]^. It increases the risk of thromboembolism by sixteen times (the incidence of stroke is 5-10% in patients with AF whereas it is 0.3% in the normal population)^[^^2^^]^. It is known that cardiovascular mortality is doubled by AF^[^^2^^]^. According to the American Heart Association (AHA), 70,000 patients a year are referred to hospitals due to AF^[^^2^^]^. Additional complications such as hemodynamic instability, palpitation, fainting, increased duration of total hospitalization, and side effects due to pharmacological treatment applied are also observed^[^^3^^]^. For this reason, the Heart Rhythm Society (HRS)/ European Heart Rhythm Association (EHRA)/ European Cardiac Arrhythmia Society (ECAS) guidelines recommend treatment of AF with ablation if cardiac surgery is performed for concomitant pathology^[^^4^^]^.

Since Cox's surgical AF ablation reported the cut-and-sew technique in 1987, only 38% of the valve replacement procedures have been coupled with AF ablation^[^^5^^]^. Although it was reported in the first years that sinus rhythm and conduction pathways were preserved, this did not relieve surgeon's concerns. In the following years, there was renewed interest among surgeons for maze procedure, after reporting that alternative sources of energy (*e.g*., radiofrequency, ultrasound and cryothermal) could create transmural lesions. The purpose of the ablation is to synchronize the atrium and the ventricles by inhibiting reentry in macroreentrant circuits and reducing the surface area to which the electrical activity of the atrium is confined, in addition to ablation of focal atrial triggers^[^^6^^]^. In order to the procedure to be effective, it is important to create transmural lesions, not disrupt the atrium functions and damage surrounding tissues^[^^7^^]^.

Although there are many studies showing the success of the Cox maze IV method, only a small number of studies comparing energy sources and focused on the causes of AF recurrence is present. Despite the initial successful results with bipolar cautery, it had disadvantages such as difficulties in handling and controlling energy, clot formation in the atrium, rupture of the atrium, and damage to surrounding tissues. Due to the risk of esophageal and coronary artery injury, in particular, the method has gradually given way to the radiofrequency ablation (RFA). Radiofrequency is a hyperthermic energy, producing transmural lesions. Alternatively, cryothermal ablation (CrA) produces lesions similar to the classical Cox maze procedure by freezing tissues and causing cellular damage at temperatures of −60/−70ºC with nitrous oxide supplementation. Despite the advantages of the formation of transmural lesions and low risk of perforation and thromboembolism, the length of the application period is a disadvantage.

In our study, we investigated the maintenance rate of postoperative sinus rhythm and the factors affecting it in cases involving concomitant AF ablation with mitral valve surgery and tried to determine whether there is a causal relationship with the energy source (RFA and CrA) used. We also tried to determine the cumulative effect of the risk factors on the maintenance of sinus rhythm and their contribution to appropriate case selection.

## METHODS

### Patients

The study was planned as a retrospective case-control study with the approval of our institution's ethics committee. The study included 96 patients who underwent either RFA or CrA procedures concomitant with mitral valve replacement and tricuspid valve repair between June 2014 and May 2018. The cases were randomly selected among patients with preoperative AF rhythm at least for three months (persistent AF) without previous history of ablation and underwent mitral and/or tricuspid valve surgery, concomitantly with surgical AF ablation. Cases with a history of such diseases leading to dysrhythmia through electrolyte irregularities as diabetes mellitus, thyroid and renal dysfunction, and cases of infective endocarditis requiring complex treatment were excluded. In the study, information from the hospital patient database and the patient's telephone records were used. The operations were performed by three different primary surgeons. All operations were performed under cardiopulmonary bypass. Cases requiring additional intervention were excluded from the study. The mean age of the cases was 50.1 (Max-Min = 27-68; 52.1% female). In 89 cases, median sternotomy was performed whereas right mini-thoracotomy was the approach of choice in seven cases. Electrocardiogram (ECG) and/or Holter recordings obtained at follow-up visits performed on the 1^st^, 3^rd^, 6^th^ and 15^th^ postoperative days and 12^th^ postoperative month by patients following one-week hospitalization were used to investigate the AF recurrence. A 24-hour Holter monitoring was performed in cases with a 6-month interval. At the control visits, the presence of acute inflammation, infection and related complications were evaluated with blood tests and physical examination (Table 1).

**Table 1:** Demographical characteristics of the cases

		Ablation type				
		RFA (n=54)		CrA (n=42)		
		Mean	SD	Mean	SD	*P*-value
Age (years)		48	9	53	9	0.016^a^
Left atrium diameter (mm)		48.98	5.45	49.05	5.72	0.954^a^
Intensive Care Unit (days)		1	1	1	__	0.145^a^
Hospitalization (days)		6	1	6	1	0.509^a^
Aortic cross-clamping time (min)		63	12	68	11	0.035^a^
Total cardiopulmonary bypass time (min)		66	10	83	10	0.001^a^
Left ventricular ejection fraction (%)		48.78	6.52	50.07	6.13	0.325^a^
Preoperative CRP (mg/dL)		4.6	1.6	4.5	1.5	0.864^a^
		**n**	**%**	**n**	**%**	
Body mass index (kg/m^2^)	<25	25	54.3	21	45.7	0.395^a^
	25-29	20	55.6	16	44.4	
	30-35	6	54.5	5	45.5	
	>35	3	100	__	__	
Gender	Female	24	48	26	52	0.089^b^
	Male	30	65.2	16	34.8	
Surgical procedure	MVR	29	65.9	15	34.1	0.079^b^
	MVR+TDVGA	25	48.1	27	51.9	
Previous stroke	Unavailable	52	55.9	41	44.1	0.712^b^
	Available	2	66.7	1	33.3	
Postoperative pacemaker	Unavailable	53	55.8	42	44.2	0.375^b^
	Available	1	100	__	__	
Chronic obstructive pulmonary disease	Unavailable	47	54.7	39	45.3	0.356^b^
	Available	7	70	3	30	
New York Heart Association functional class	I	10	41.7	14	58.3	0.13^a^
	II	30	60	20	40	
	III	14	63.6	8	36.4	
	IV	__	__	__	__	
Hypertension	Unavailable	45	55.6	36	44.4	0.75^b^
	Available	9	60	6	40	

a =independent t-test;

b =Chi-square test; Fisher's exact test, continuity correction test.

CrA=cryoablation; MVR+TDVGA=mitral valve replacement+tricuspid De Vega annuloplasty; RFA=radiofrequency ablation; SD=standard deviation

### Ablation Method

**Fig. 1:**
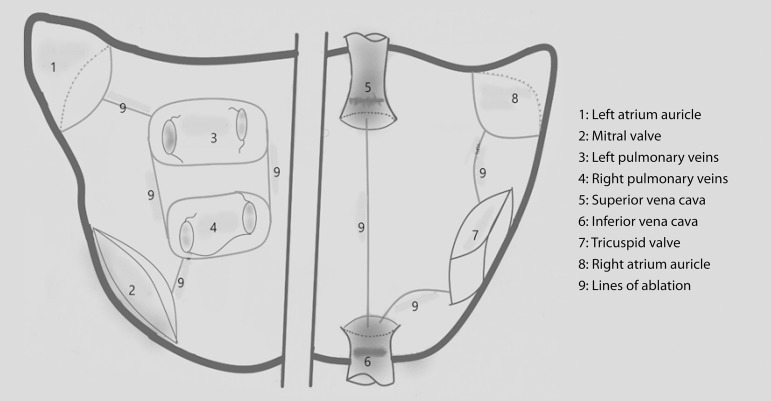
Schematic drawing on ablation technique used in the study.

Cryothermal energy (AtriCure^®^ cryoICE BOX surgical ablation system, model cryoICE BOX2-230 VAC) was used as an energy source for ablation in 42 (43.8%) patients, while bipolar radiofrequency energy was used in 54 (56.3%) patients (AtriCure^®^ Articulating Jaw, Isolator Synergy Access^®^). In all cases, the biatrial ablation technique shown in Figure 1 was applied. Access to atrioventricular valves and/or atria done through superior septal incision in the cases with median sternotomy, while in those with the thoracotomy was performed through the separate left and right atriotomies. Because of the ease of manipulation, CrA was preferred in cases of thoracotomy. Ablation procedure was performed based on the incision lines described in Figure 1. The same procedure was preferred for both cryothermal and radiofrequency ablation. Ablation was performed prior to valve replacement and division of the left atrial appendage. The left atrial appendix was closed using 4.0 Prolene sutures. Ablation procedure took 10-15 min for RFA and 25-30 min for CrA.

### Follow-up Protocol

In the first 3 postoperative days, all cases were followed by continuous ECG monitoring. Perioperative amiodarone hydrochloride infusion (intravenous in 5% dextrose, 10-15 mg/kg/24h) was started according to the protocol applied in our clinic. Treatment with amiodarone hydrochloride (200 mg twice a day postoperative) and indomethacin (25 mg twice a day p.o.) was continued for 3 months in patients with oral intake. Three months later, metoprolol tartrate therapy (100 mg once a day postoperative) was started. During an average of 7 days of hospitalization, the heart rate was followed by daily ECG recording. Patients were called for control visits on the 1^st^, 3^rd^, 6^th^ and 15^th^ postoperative days and 12 months after discharge. The patients were examined in terms of inflammation, infection, stroke and rhythm disturbances, as well as the need for further treatment. Rhythm follow-up was done with ECG recording in the routine with Holter monitoring in the 6^th^ and 12^th^ months. In cases with permanent pacemaker insertion due to complete atrioventricular block developed postoperatively, pacemaker follow-up was performed. Antiarrhythmic therapy of patients with a heart rate below 60 beats/min was discontinued. In patients with recurrent AF, antiarrhythmic therapy was restarted and 24-hour ECG monitoring was performed. Electrical cardioversion was applied to cases detected early. Patients who did not respond to maximal antiarrhythmic treatment and electrical cardioversion were referred to cardiology for catheter ablation

### Statistical Analysis

Statistical Package for Social Sciences Statistical Software version 18.0 (SPSS Inc., Chicago, Il, USA) was used in our single-center retrospective case-control study. Continuous variables were expressed as mean and standard deviation, while Student t-test was used for the comparisons. Paired samples t-test was used in the analysis of the dependent data. Categorical and nominal variables were expressed in terms of number and percentage (%). The relationship status was determined by chi-square, Fisher's exact test, and continuity correction tests. Multinomial logistic and linear regression analyzes were used to determine the factors affecting maintenance of sinus rhythm by analyzing age, gender, left ventricular ejection fraction (LVEF), the presence of chronic obstructive pulmonary disease (COPD), type of energy source, length of hospital stay, body mass index (BMI), left atrial diameter, cross-clamping time and C-reactive protein (CRP) levels. The cut-off points of risk factors detected in cases with postoperative AF recurrence were determined by receiver operating characteristic (ROC) analysis. The cumulative effect of AF at one year and the significance level by months were tested by Kaplan-Meier survey analysis. The results were considered significant when the two-sided *P*-value was <0.05.

## RESULTS

**Fig. 2:**
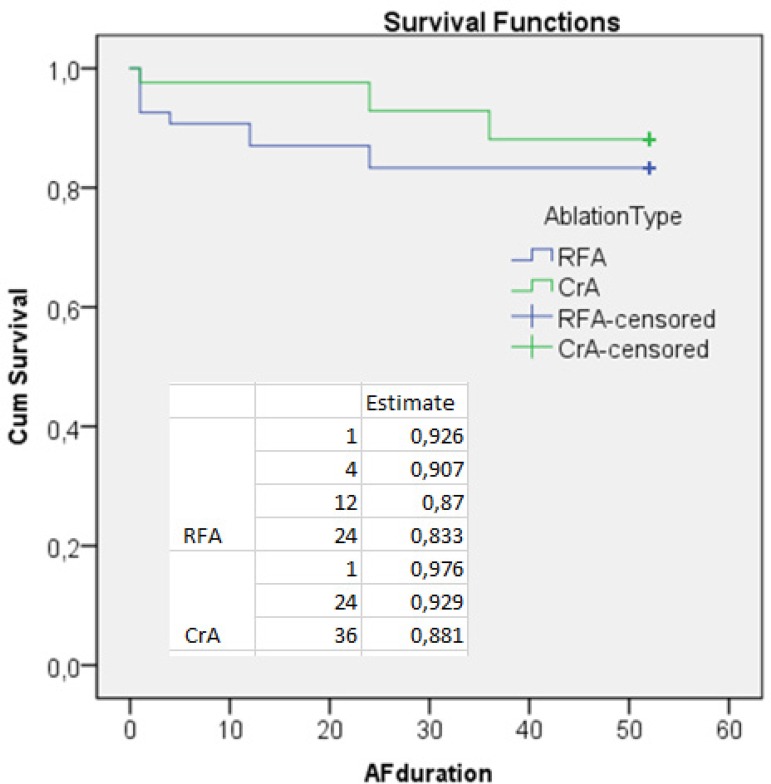
Survival analysis of cases according to the energy source used. AF=atrial fibrillation; CrA=cryothermal ablation; RFA=radiofrequency ablation

Bipolar RFA was used in 54 patients (56.3%, 24 females) while CrA was used in 42 patients (43.8%, 26 females). There were no significant differences between the demographic characteristics of the groups, except age and cross-clamping time. Distribution of demographic and operative characteristics is shown in Table 1. Maintenance rates of sinus rhythm in the CrA and RFA groups were 97.6% and 96.3% in the early postoperative period, and 88.1% and 83.3% in the 12^th^ postoperative month (mean 85.4%), respectively. No significant difference was found between the groups in relation to the energy source used in the early postoperative period and after 12 months (Mentel-Cox *P*=0.455; Figure 2). There was no mortality during follow-up. In the CrA group, postoperative transient cerebral ischemic attack was observed in one (2.3%) case. In the bipolar RFA group, perioperative posterior wall rupture was observed in one (1.8%) case, hemiplegia in one (1.8%) case and permanent pacemaker need in one (1.8%) case. The wall rupture was sutured without additional complications.

**Fig. 3:**
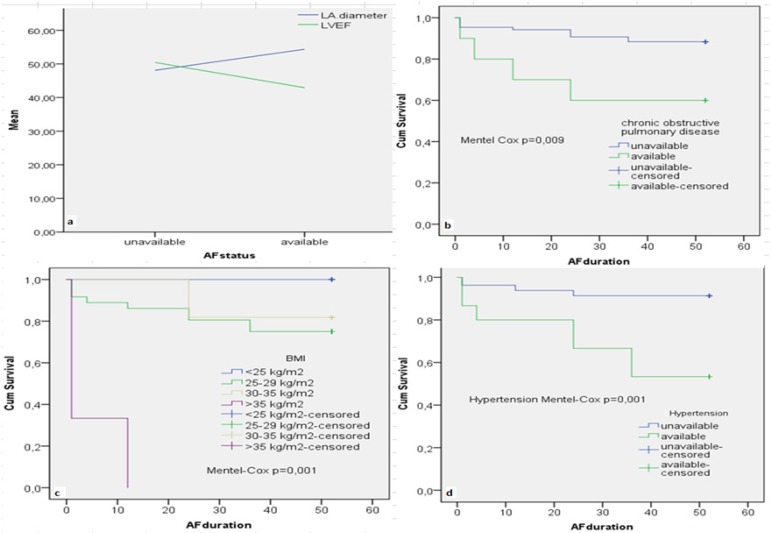
a) Change in the recurrence of AF with left atrium diameter and LVEF. b, c, d) Survival analysis of postoperative COPD, BMI and hypertension with Mentel-Cox significance values. BMI=body mass index; COPD=chronic obstructive pulmonary disease; LA= eft atrium; LVEF=left ventricular ejection fraction

**Fig. 4:**
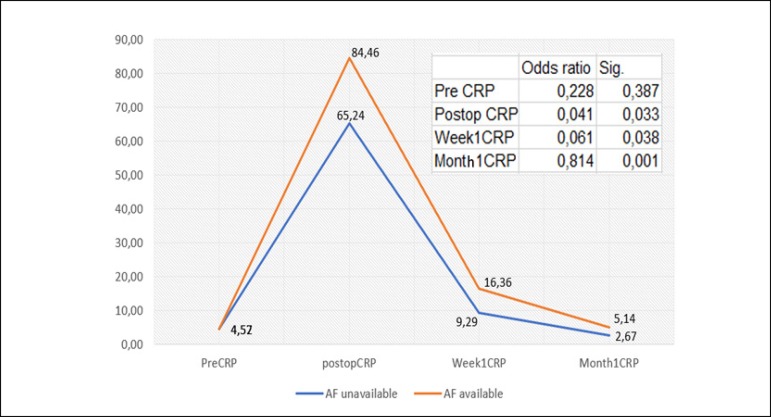
The change in CRP levels in relation to the cases with and without AF recurrence and the significance ratios according to Cox regression analysis. CRP=C-reactive protein

**Fig. 5:**
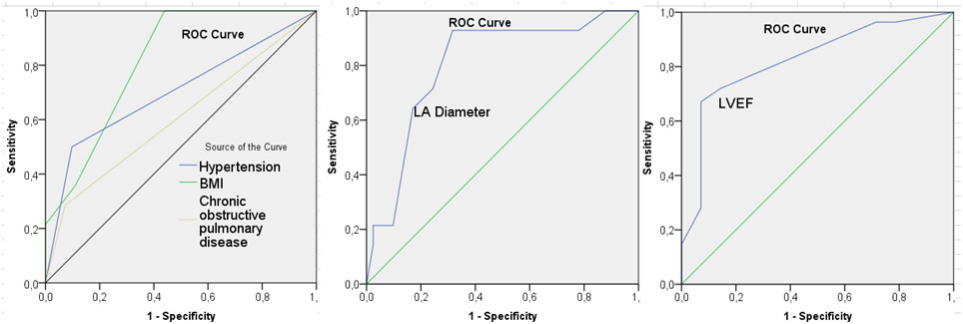
ROC curve analysis graphs of factors affecting AF recurrence. AF=atrial fibrillation; BMI=body mass index; LA=left atrium; LVEF=left ventricular ejection fraction

**Fig. 6:**
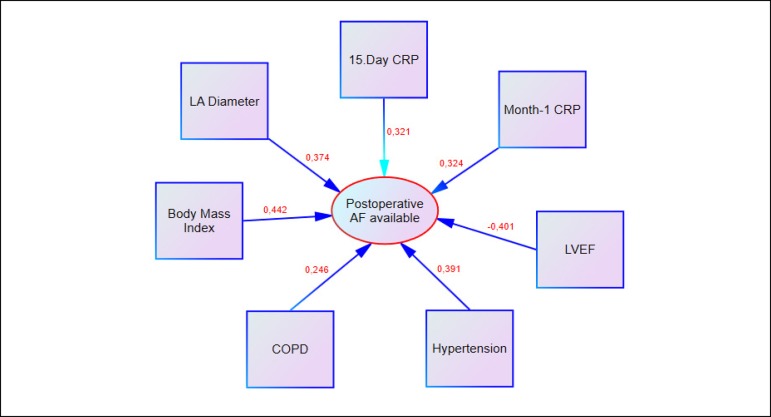
Factors correlated with AF recurrence after ablation (r correlation coefficient of significance at P<0.05 were taken). AF=atrial fibrillation; COPD=chronic obstructive pulmonary disease; CRP=C-reactive protein; LA=left atrium; LVEF=left ventricular ejection fraction

In the postoperative follow-up, the causal relationship between AF recurrence and left atrial diameter, CRP level, hypertension, LVEF, COPD and BMI was found (Table 2; Figures 3-6; *P*<0.05).

**Table 2:** Distribution of risk parameters in relation to postoperative atrial fibrillation status.

		AF status				
		Unavailable		Available		*P*-value
		Mean	SD	Mean	SD	
Age (years)		50	9	50	10	0.921^a^
Left atrium diameter (mm)		48.1	5.11	54.36	5.06	0.001^a^
Intensive Care Unit (days)		1	1	1	1	0.685^a^
Hospitalization (days)		6	1	6	1	0.843^a^
Aortic cross-clamping time (min)		64	11	68	14	0.236^a^
Cardiopulmonary bypass time (min)		75	13	68	16	0.08^a^
Left ventricular ejection fraction (%)		50.44	5.85	42.93	5.46	0.001^a^
		**n**	**%**	**n**	**%**	
Body mass index (kg/m^2^)	<25	46	100	__	__	0.001^a^
	25-29	27	75	9	25	
	30-35	9	81.8	2	18.2	
	>35	__	__	3	100	
Gender	Woman	43	86	7	14	0.872^b^
	Man	39	84.8	7	15.2	
Surgical procedure	MVR	35	79.5	9	20.5	0.137^b^
	MVR+TDVGA	47	90.4	5	9.6	
Previous stroke	Unavailable	79	84.9	14	15.1	0.472^b^
	Available	3	100	__	__	
Postoperative pacemaker	Unavailable	81	85.3	14	14.7	0.682^b^
	Available	1	100	__	__	
Chronic obstructive pulmonary disease	Unavailable	76	88.4	10	11.6	0.016^b^
	Available	6	60	4	40	
New York Heart Association functional class	I	23	95.8	1	4.2	0.343^a^
	II	40	80	10	20	
	III	19	86.4	3	13.6	
	IV	__	__	__	__	
Hypertension	Unavailable	74	91.4	7	8.6	0.001^b^
	Available	8	53.3	7	46.7	
Ablation type	RFA	45	83.3	9	16.7	0.517^b^
	CrA	37	88.1	5	11.9	
CRP^1^ (mg/dL)	Postoperative	65.2	26.4	84.5	7.5	0.266^a^
	15^th^ postoperative day	9.3	6.9	16.4	8.9	0.003^a^
	1^st^ postoperative month	2.7	1.2	5.1	1.0	0.001^a^

a =independent t test;

b =Chi-square, Fisher's exact test, continuity correction test

1 =for statistical analysis of AF recurrence, the data on the same day and month were taken into consideration.

MVR+TDVGA=mitral valve replacement +tricuspid De Vega annuloplasty; SD=standard deviation; AF=atrial fibrillation; CrA=cryothermal ablation; CRP=C-reactive protein; MVR=mitral valve replacement; RFA=radiofrequency ablation

There was a moderate correlation (37.4%) between left atrial diameter and AF recurrence (*P*=0.001; r=0.374; Figure 3). Sensitivity and specificity of left atrial diameter with a cut-off value of 50.5 mm for the maintenance of the sinus rhythm were 85.7% and 70.7%, respectively (area=0.805; *P*=0.001; 95% CI=0.687-0.923; Figure 5).

The mean preoperative CRP value was 4.5 mg/dL. There was a moderate positive correlation between AF recurrence and CRP level on the 15^th^ postoperative day and at the 1^st^ postoperative month (*P*=0.001; r=0.321 and *P*=0.001; r=0.324, respectively; Figure 3D), whereas no correlation was found between AF recurrence and CRP on the 1^st^ postoperative day (*P*=0.193; r=0.134). In the ROC analysis, the CRP level on the 15^th^ postoperative day with a cut-off value of 12 mg/dL showed sensitivity of 83.3% and specificity of 88.9% (area=0.873; *P*=0.002; 98% CI=0.804-0.942). Sensitivity and specificity of CRP level at a cut-off value of 4.5 mg/dL for the maintenance of the sinus rhythm at 1^st^ postoperative month were 66.7% and 87.8%, respectively (area=0.879; *P*=0.046, 95% CI = 0.789-0.968; Table 1; Figure 5).

As BMI values increased, the AF recurrence rate increased significantly. The increase in BMI affected AF recurrence by 3.2 times (*P*=0.001; r=0.442; Figures 3 and 5). In cases with BMI>30 kg/m^2^ (14.6%; 14 cases), recurrent AF frequency (35.7%; 5 cases) was significant (Table 2). In the ROC analysis, the sensitivity of the BMI at 30 kg/m^2^ cut-off was 35.7%, while the specificity was 89% (area=0.816; *P*=0.001; 95% CI=0.720-0.911).

The mean value of LVEF was 49.3% (35-60%). As LVEF decreased, AF recurrence increased (40.1%), indicating a moderate inverse correlation (*P*=0.001; r=−0.401). Sensitivity at 37.5% cut-off value of LVEF in ROC analysis was 96.3%, while the specificity was 11.4% (area=0.825; *P*=0.001; 95% CI=0.711-0.939; Figures 3 and 5).

It was found that 15.7% of all cases had hypertension. Considering the prevalence of hypertension in patients with AF (50%), this ratio was nonspecific in our study, which included only valvular patients. AF recurrence was seen in 7 of 15 (46.7%) hypertensive patients. Hypertension affected postoperative AF recurrence by 5.3 times (*P*=0.001; r=0.391; Figure 4; OR=0.192; 96% CI=0.084-0.453). Patients with COPD accounted for 10.4% of our cases. Recurrent AF was observed in 4 (40%) cases with COPD. Low correlation with AF recurrence was detected (*P*=0.016; r=0.246; Figure 5).

Postoperative intensive antiarrhythmic therapy was initiated in all cases and continued for 3 months. Electrical cardioversion was applied in 3 (3.1%) cases in one year. Additional catheter ablation was performed for 2 (14%) of 14 (14.1%) patients who developed AF at annual follow-up. No mortality was observed.

## DISCUSSION

From 1987 to 1992, when the Cox maze procedure was reported for the first time, continuity of sinus rhythm was reported to be 85-98% in the Cox maze III procedure, which was developed through experiments with dog models^[^^7^^]^. In a meta-analysis involving six studies, the maintenance rate of sinus rhythm in a one-year follow-up of 97 cases undergoing mitral valve replacement was reported as being 44% for those undergoing concomitant RFA and 4.5% for those without RFA^[^^8^^]^. In a meta-analysis involving randomized and non-randomized controlled trials, Barnett and Ad^[^^9^^]^ reported higher rates of sinus rhythm restoration (94%) in patients undergoing AF ablation in addition to cardiac surgery than in those patients undergoing cardiac surgery alone (4.4%). The limiting characteristic of the original cut-and-sew technique was the fear of surgeons arising from the complexity of the procedure, as well as the increased possibility of complete atrioventricular block and other complications. For this reason, the Cox maze IV procedure, which produces transmural lesions with different energy sources, was developed. In a study by Lall et al.^[^^10^^]^, maintenance rates of sinus rhythm for one year follow-up after Cox maze III and IV procedures were found to be 96% and 93%, respectively. The success rate of concomitantly performed AF ablations was 67-84% in retrospective reports^[^^11^^]^. The variation in the studies was due to different lesion lines, different sources of energy, surgeon experience and follow-up strategies. In our study, the maintenance rate of sinus rhythm was 85.4% at one-year follow-up. Our results were similar to those of the literature. It is understood from the studies performed by McCarthy et al.^[^^12^^]^ that there are significant differences in quality of life among patients with AF who underwent simultaneous AF ablation with valve replacement compared with patients who had valve replacement without AF ablation.

Bipolar RFA and CrA are the most frequently recommended techniques for ablation during mitral valve replacement^[^^13,14^^]^. Bipolar RFA works according to the principle of lesion formation through a transmural hyperthermic heat exchanger clamp in endocardial and epicardial tissues. Feedback information on whether these lesions were transmural or not was effective in the transition from unipolar to bipolar systems. Bipolar RFA devices have been used in our hospital since 2007, but after 2014, nitrous oxide-based CrA devices were preferred. Cryotherapy works through the Joule-Thomson effect, cooling the tissues. The intracellular ice formation, which can be monitored intraoperatively, results in necrosis of the cell^[^^13^^]^. Transmural lesion with CrA was demonstrated histologically in experimental sheep models in endocardial and epicardial applications^[^^14^^]^. It has advantages such as visual confirmation of ice formation, formation of progressive transmural lesions, low risk of injury to neighboring tissues and no reports of injuries in the valve leaflet, phrenic nerve and coronary arteries provided in the literature, even though it is relatively early. The length of the application is the disadvantage of CrA, which has been shown to have no effect on morbidity and mortality^[^^15^^]^. Brick et al.^[^^15^^]^ reported in their meta-analysis of 19 articles that found no difference between the one year success rates of sinus rhythm maintaining after RFA (67-96%) and CrA (65.5-97.7%). In concomitant interventions, the maintenance rate of one year sinus rhythm was 83.3% for bipolar RFA and 88.1% for CrA (Figure 2). However, the effect of energy sources on AF recurrence was not significant.

Although Cox maze IV procedure is applicable in both atriums, there are publications in recent years reporting that only left atrial ablation could have been sufficient^[^^16^^]^. In a study by Gillinov et al.^[^^17^^]^ comparing biatrial AF ablation with left atrial AF ablation, the biatrial ablation technique was significantly superior to the other. However, in the studies of Worku et al.^[^^18^^]^, the rate of persistent pacemaker implantation after biatrial AF ablation was reported as significantly higher. In 268 cases of biatrial cryothermal Cox maze IV procedure performed by Funatsu et al.^[^^19^^]^, the rate of sinus rhythm restoration in permanent AF was reported to be 80.2%, while the need for permanent pacemaker was 8.3%. The need for permanent pacemaker can be explained by the preoperative undetected sinus node dysfunction of patients with AF. The incidence of pacemaker implantation is also high in the routine follow-up of these cases^[^^20^^]^. The fact is that surgical ablation performed only in the left atrium may be satisfactory, but the increase in flatter incidence from the right atrium after surgery is inevitable. For this reason, biatrial ablation should be preferred, especially in cases with a history of the atrial flutter^[^^19^^]^. Permanent pacemaker requirement was seen in only one (1.8%) of our patients who underwent RFA. Our rate of sinus rhythm per year was similar to that of the literature. Our opinion is that it is correct for the surgeon to make a case-based decision on the method of ablation.

Bipolar RFA is not used in minimally invasive procedures because of the difficulties encountered in the complete realization of Cox's maze lesions and its ability to allow only the pulmonary vein isolation, which we refer to as "box lesions". However, CrA is more advantageous in minimally invasive procedures, since the tip of the catheter used can be put in any desired shape.

Analyzes have shown that ablation does not increase operative mortality, and, on the contrary, reduces late mortality and morbidity since it reduces postoperative thromboembolic risk^[^^15^^]^. Phan et al.^[^^20^^]^ found no significant difference in postoperative stroke and mortality between two groups of patients, one with concomitant AF ablation and the other without it. There was no mortality in our study. One (1.8%) case had a stroke in the late period. Gillinov et al.^[^^17^^]^ reported 3% incidence of stroke for one-year follow-up. Our stroke rate was much lower than the risk of AF induced thromboembolism (10-20%), even if the risk of valve thrombosis was added. Despite the prolonged duration of cardiopulmonary bypass, there was no difference in terms of stroke and mortality in the CrA group.

Complications such as esophageal and coronary arterial damage that occurred in the first applications are gradually reduced with the introduction of new energy sources^[^^21^^]^. In a published report of esophageal perforations after surgical AF ablation, 26 of 29 cases of esophageal perforation were reported to have occurred after RFA and one case after CrA^[^^21^^]^. No esophageal perforation was detected in our cases. In only one case, there was minimal damage to the posterior wall of the left atrium after bipolar RFA. The damage was repaired without additional complications.

It is not sufficient to use only intermittent ECG records in the long-term follow-up of patients with restored sinus rhythm due of the possibility that rhythm is caught in a short and transient period of sinus node activity, and thus, a paroxysmal atrial arrhythmia may go unnoticed. For this reason, we think that it is best to use a 24-hour Holter ECG or rhythm monitor, especially in the early period. In our cases, we performed Holter monitoring every 6 months. Continuous ECG monitoring was performed in cases with recurrent AF. Therapeutic modalities such as antiarrhythmic therapy and cardioversion were applied in the recurrent AF cases detected at 12 months of follow-up.

After the operation, amiodarone and nonsteroidal anti-inflammatory drug therapy were applied for 3 months, considering the catecholamine increase and metabolic inducers. The first 3 months after ablation are called the "blind period". In this period, antiarrhythmics may prevent early AF recurrence triggered by ablated tissue-derived rhythm disorders (proarrhythmias)^[^^22^^]^. Amiodarone also provides rate control against arrhythmia.

Restoration of sinus rhythm after ablation is an independent predictor of successful ablation in the 12^th^ postoperative month. Damiano et al.^[^^23^^]^ found high rates of AF recurrence at a 12^th^ postoperative month in patients with atrial tachycardia in the early postoperative period. AF recurrence was observed in 6 of 14 (42.9%) cases in the first 15 days (Figure 2). We think that early AF recurrence is caused by the intense effect of proarrhythmic factors.

Since the early 2000's, researchers have argued that AF is caused by inflammatory processes. The study results have shown that there is a correlation between CRP and inflammation^[^^24^^]^. However, the effect of inflammation on the left atrium size and the pathogenesis of the dysrhythmias is controversial. Psychari et al.^[^^24^^]^ found a significant association between AF occurrence and CRP and interleukin-6 levels in their study of 90 patients. In their study of 50 patients with and without persistent AF, Watanabe et al.^[^^25^^]^ reported that the left ventricular mass and increased left ventricular end-diastolic diameter were determinants of CRP elevation and AF persistence. In our study, CRP levels were found to be high but not significant on the 1^st^ postoperative day. On the 15^th^ postoperative day, the sensitivity and specificity of CRP in terms of effect on AF recurrence with a cut-off value of 12 mg/dL were found to be 83.3% and 88.9%, respectively (area=0.873; *P*=0.002; 98% CI=0.804-0.942; Figures 2-4). The sensitivity and specificity ratios at 1^st^ postoperative month for CRP with a cut-off value of 4.5 mg/dL were 66.7% and 87.8%, respectively (area=0.879; P=0.046; 95% CI=0.789-0.968; Table 1). The effect of energy sources on postoperative CRP level was not significant (*P*>0.05; Table 1).

Funatsu et al.^[^^19^^]^ reported that AF recurrence rate after ablation was increased when the left atrium size was ≥70 mm and AF had been present over 10 years. Some studies have reported that left atrium diameter greater than 60 mm and the presence of hypertension are independent risk factors for unsuccessful ablation^[^^23^^]^. Although the effect of left atrium diameter on primary AF is supported, there is insufficient data on whether left atrium diameter after ablation is a predictor for AF recurrence. In our study, sensitivity and specificity ratios of left atrium diameter for AF recurrence with a cut-off value of 50.5 mm were 85.7% and 70.7%, respectively. Left atrium diameter was greater than 50.5 mm in 12 (85.7%) of the 14 patients with recurrent AF. Our results are similar to those of Chavez et al.^[^^11^^]^.

Ducceschi et al.^[^^26^^]^ reported that, in a series of 150 cases (BMI >30 kg/m^2^), AF was more common in obese patients. Adipose tissue is an active endocrine organ that secretes many hormones and cytokines (TNF-α, IL-6, IL-8), such as leptin, resistin and adiponectin. These cytokines lead to systemic inflammation and affect insulin resistance and pulmonary function^[^^24^^]^. This causes left atrium growth and contributes to irregularity in electrolyte metabolism. Recurrent AF was more frequent in our cases with postoperative BMI >30 kg/m^2^ (14 cases) and the difference was significant (Table 2; Figures 3 to 6). Sensitivity and specificity of obesity were 35.7% and 89%, respectively, in terms of AF recurrence with a cut-off value of 30 kg m^2^ (area=0.816; *P*=0.001; 95% CI=0.720-0.911). The increase in BMI affected AF recurrence by 3.2-fold.

The prevalence of hypertension in developed countries is around 25-30%^[^^27^^]^. Cohort studies showed the presence of hypertension in 53% of patients with AF and a causal linkage in 15% of patients with AF^[^^27^^]^. In our cases, there were sufficient reasons for the development of AF, but 15 patients were being treated for hypertension at the same time (Table 1). Recurrence of postoperative AF was seen in 7 (46.7%) cases with hypertension. The recurrence of AF was 5.3 times more frequent in hypertensive cases when compared to non-hypertensive cases.

Our experience and the studies reported in the literature have shown that successful results are obtained if AF ablation is carried out during cardiac surgery. Although there are no clear criteria for patient selection, cases with left atrial diameter ≥50 mm, LVEF ≤ 37%, and heart failure of NYHA class III-IV should be excluded. Nevertheless, in the American College of Cardiology (ACC)/AHA and European Society of Cardiology (ESC)/EHRA guidelines^[^^4^^]^, AF ablation is recommended with a low level of evidence (Class 2b) for cases with medical treatment refractory to symptomatic heart failure and/or atrial enlargement. In our cases, LVEF presented 96.3% sensitivity and 11.4% specificity with a cut-off value of 37.5% (area=0.825; *P*=0.001; 95% CI 0.711-0.939; Figures 3 and 5).

### Limitation

Detection of recurrent AF in a longer period may not be possible, since our follow-up period has been limited to 1 year. For this reason, we realize that the most valuable results for the comparison of different energy sources used in Cox maze IV procedure will come from large series with long-term follow-up.

In our single-center nonrandomized trial, it was not possible to eliminate the confounding variables that affect AF recurrence. For this reason, our 1-year follow-up was influenced by factors other than energy sources. However, it is understood from the demographic data that the confounding factors that we have detected do not display nonuniform distribution among the groups (Table 1).

## CONCLUSION

Considering the high incidence and complication rate, persistent AF requires aggressive treatment and follow-up, we believe that it is necessary to perform AF ablation in case of necessity in all cases where cardiac surgery is planned. In our cases with bipolar RFA and CrA, we found acceptable rates of AF recurrence, mortality and stroke at one-year follow-up. Even if there is no difference between the efficiency of the energy systems we used, an increase in the demand for alternative systems will occur in a world moving toward less invasive procedures. We believe that controlling risk factors and following appropriate medical procedures are as effective as surgical procedure and energy source on sinus rhythm maintenance. However, we believe that if cardiologists and surgeons interested in electrophysiology work together with a multidisciplinary team approach in choosing the right patient and procedure, success rate first and then quality of life will increase.

**Table:** 

**Authors’ roles & responsibilities**	
ÜV	Substantial contributions to the conception or design of the work; or the acquisition, analysis, or interpretation of data for the work; drafting the work or revising it critically for important intellectual content; agreement to be accountable for all aspects of the work in ensuring that questions related to the accuracy or integrity of any part of the work are appropriately investigated and resolved; final approval of the version to be published
AYB	Final approval of the version to be published
AAA	Agreement to be accountable for all aspects of the work in ensuring that questions related to the accuracy or integrity of any part of the work are appropriately investigated and resolved; final approval of the version to be published
MK	Drafting the work or revising it critically for important intellectual content; final approval of the version to be published

## References

[1] Camm AJ, Kirchhof P, Lip GY, Schotten U, Savelieva I, Ernst S, ESC Committee for Practice Guidelines (2010). Guidelines for the management of atrial fibrillation: the Task
Force for the Management of Atrial Fibrillation of the European Society of
Cardiology (ESC). Europace.

[2] Fornari LS, Calderaro D, Nassar IB, Lauretti C, Nakamura L, Bagnatori R (2007). Misuse of antithrombotic therapy in atrial fibrillation patients:
frequent, pervasive and persistent. J Thromb Thrombolysis.

[3] Adalet K (2002). The surgical treatment of atrial fibrillation. Türk Kardiyol Dern Arfl.

[4] Calkins H, Kuck KH, Cappato R, Brugada J, Camm AJ, Chen SA, Heart Rhythm Society Task Force on Catheter and Surgical Ablation of
Atrial Fibrillation (2012). 2012 HRS/EHRA/ECAS expert consensus statement on catheter and
surgical ablation of atrial fibrillation: recommendations for patient
selection, procedural techniques, patient management and follow-up,
definitions, endpoints, and research trial design: a report of the Heart
Rhythm Society (HRS) Task Force on Catheter and Surgical Ablation of Atrial
Fibrillation. Developed in partnership with the European Heart Rhythm
Association (EHRA), a registered branch of the European Society of
Cardiology (ESC) and the European Cardiac Arrhythmia Society (ECAS); and in
collaboration with the American College of Cardiology (ACC), American Heart
Association (AHA), the Asia Pacific Heart Rhythm Society (APHRS), and the
Society of Thoracic Surgeons (STS). Endorsed by the governing bodies of the
American College of Cardiology Foundation, the American Heart Association,
the European Cardiac Arrhythmia Society, the European Heart Rhythm
Association, the Society of Thoracic Surgeons, the Asia Pacific Heart Rhythm
Society, and the Heart Rhythm Society. Heart Rhythm.

[5] Gammie JS, Haddad M, Milford-Beland S, Welke KF, Ferguson Jr TB, O'Brien SM (2008). Atrial fibrillation correction surgery: lessons from the Society
of Thoracic Surgeons National Cardiac Database. Ann Thorac Surg.

[6] Gillinov AM, Sirak J, Blackstone EH, McCarthy PM, Rajeswaran J, Pettersson G (2005). The Cox maze procedure in mitral valve disease: predictors of
recurrent atrial fibrillation. J Thorac Cardiovasc Surg.

[7] Cox JL, Ad N, Palazzo T, Fitzpatrick S, Suyderhoud JP, DeGroot KW (2000). Current status of the Maze procedure for the treatment of atrial
fibrillation. Semin Thorac Cardiovasc Surg.

[8] Doukas G, Samani NJ, Alexiou C, Oc M, Chin DT, Stafford PG (2005). Left atrial radiofrequency ablation during mitral valve surgery
for continuous atrial fibrillation: a randomized controlled
trial. JAMA.

[9] Barnett SD, Ad N (2006). Surgical ablation as treatment for the elimination of atrial
fibrillation: a meta-analysis. J Thorac Cardiovasc Surg.

[10] Lall SC, Melby SJ, Voeller RK, Zierer A, Bailey MS, Guthrie TJ (2007). The effect of ablation technology on surgical outcomes after the
Cox-maze procedure: a propensity analysis. J Thorac Cardiovasc Surg.

[11] Chavez EK, Colafranceschi AS, Monteiro AJO, Canale SC, Mesquita ET, Weksler C (2017). Surgical treatment of atrial fibrillation in patients with
rheumatic valve disease. Braz J Cardiovasc Surg.

[12] McCarthy PM, Manjunath A, Kruse J, Andrei AC, Li Z, McGee Jr EC (2013). Should paroxysmal atrial fibrillation be treated during cardiac
surgery?. J Thorac Cardiovasc Surg.

[13] Gage AA, Baust J (1998). Mechanism of tissue injury in cryosurgery. Cryobiology.

[14] Gallegos RP, Rivard AL, Rajab TK, Schmitto JD, Lahti MT, Kirchhof N (2011). Transmural atrial fibrosis after epicardial and endocardial
argon-powered CryoMaze ablation. J Card Surg.

[15] Brick AV, Braile DM (2015). Surgical ablation of atrial fibrillation using energy
sources. Braz J Cardiovasc Surg.

[16] Khargi K, Deneke T, Lemke B, Laczkovics A (2003). Irrigated radiofrequency ablation is a safe and effective
technique to treat chronic atrial fibrillation. Interact Cardiovasc Thorac Surg.

[17] Gillinov AM, Gelijns AC, Parides MK, DeRose Jr JJ, Moskowitz AJ, Voisine P, CTSN Investigators (2015). Surgical ablation of atrial fibrillation during mitral-valve
surgery. N Engl J Med.

[18] Worku B, Pak SW, Cheema F, Russo M, Housman B, Van Patten D (2011). Incidence and predictors of pacemaker placement after surgical
ablation for atrial fibrillation. Ann Thorac Surg.

[19] Funatsu T, Kobayashi J, Nakajima H, Iba Y, Shimahara Y, Yagihara T (2009). Long-term results and reliability of cryothermic ablation based
maze procedure for atrial fibrillation concomitant with mitral valve
surgery. Eur J Cardiothorac Surg.

[20] Phan K, Xie A, La Meir M, Black D, Yan T (2014). Surgical ablation for treatment of atrial fibrillation in cardiac
surgery: a cumulative meta-analysis of randomized controlled
trials. Heart.

[21] Singh SM, d'Avila A, Singh SK, Stelzer P, Saad EB, Skanes A (2013). Clinical outcomes after repair of left atrial-esophageal fistulas
occurring after atrial fibrillation ablation procedures
Heart. Rhythm.

[22] Cosedis Nielsen J, Johannessen A, Raatikainen P, Hindricks G, Walfridsson H, Kongstad O (2012). Radiofrequency ablation as initial therapy in paroxysmal atrial
fibrillation. N Engl J Med.

[23] Damiano Jr RJ, Schwartz FH, Bailey MS, Maniar HS, Munfakh NA, Moon MR (2011). The Cox maze IV procedure: predictors of late
recurrence. J Thorac Cardiovasc Surg.

[24] Psychari SN, Apostolou TS, Santos L, Hamodraka E, Liakos G, Kremastinos DT (2005). Relation of elevated C-reactive protein and interleukin-6 levels
to left atrial size and duration of episodes in patients with atrial
fibrillation. Am J Cardiol.

[25] Watanabe T, Takeishi Y, Hirono O, Itoh M, Matsui M, Nakamura K (2005). C-reactive protein elevation predicts the occurrence of atrial
structural remodeling in patients with paroxysmal atrial
fibrillation. Heart Vessels.

[26] Ducceschi V, D'Andrea A, Liccardo B, Alfieri A, Sarubbi B, De Feo M (1999). Perioperative clinical predictors of atrial fibrillation
occurrence following coronary artery surgery. Eur J Cardiothorac Surg.

[27] Verdecchia P, Angeli F (2005). Natural history of hypertension subtypes. Circulation.

